# Multi-compartment microscopic diffusion imaging

**DOI:** 10.1016/j.neuroimage.2016.06.002

**Published:** 2016-06-06

**Authors:** Enrico Kaden, Nathaniel D. Kelm, Robert P. Carson, Mark D. Does, Daniel C. Alexander

**Affiliations:** a Centre for Medical Image Computing, University College London, UK; b Institute of Imaging Science, Vanderbilt University, USA; c Departments of Neurology and Pediatrics, Vanderbilt University, USA

**Keywords:** Spherical Mean Technique (SMT), Microscopic diffusion anisotropy, Neurite density, Fibre crossings, Orientation dispersion, Tuberous Sclerosis Complex (TSC)

## Abstract

This paper introduces a multi-compartment model for microscopic diffusion anisotropy imaging. The aim is to estimate microscopic features specific to the intra- and extra-neurite compartments in nervous tissue unconfounded by the effects of fibre crossings and orientation dispersion, which are ubiquitous in the brain. The proposed MRI method is based on the Spherical Mean Technique (SMT), which factors out the neurite orientation distribution and thus provides direct estimates of the microscopic tissue structure. This technique can be immediately used in the clinic for the assessment of various neurological conditions, as it requires only a widely available off-the-shelf sequence with two *b*-shells and high-angular gradient resolution achievable within clinically feasible scan times. To demonstrate the developed method, we use high-quality diffusion data acquired with a bespoke scanner system from the Human Connectome Project. This study establishes the normative values of the new biomarkers for a large cohort of healthy young adults, which may then support clinical diagnostics in patients. Moreover, we show that the microscopic diffusion indices offer direct sensitivity to pathological tissue alterations, exemplified in a preclinical animal model of Tuberous Sclerosis Complex (TSC), a genetic multi-organ disorder which impacts brain microstructure and hence may lead to neurological manifestations such as autism, epilepsy and developmental delay.

## Introduction

In biomedical research and clinical practice, diffusion MRI is today’s method of choice for the noninvasive detection of microscopic tissue structure below the nominal image resolution. This technique provides measurements that are sensitive to diagnostically relevant features in the range of few micrometres like cell size, shape and density. Diffusion tensor imaging (DTI), a popular method which builds on a second-order approximation of the macroscopic diffusion process (Basser et al., 1994), is routinely used for the clinical assessment of pathological changes in the brain microstructure. However, the DTI-based anisotropy indices are not only a function of microscopic tissue features, but are confounded by fibre crossings and orientation dispersion, which are ubiquitous in the brain (Schmahmann and Pandya, 2006). Consequently, it is difficult to trace the origin of observed signal abnormalities; whether they are due to intrinsic alterations in the tissue microstructure or are caused by deviations in the neural circuitry which have modified the neurite orientation distribution. Any comparisons of DTI anisotropy metrics between subjects are affected by the brain connectome, which exhibits both high complexity and interindividual variability.

The directional tissue architecture, including fibre crossings and orientation dispersion, can be accurately described by orientation distributions. The spherical convolution with the microscopic diffusion signal (also known as impulse response function), which denotes the signal arising from a potentially anisotropic microenvironment of nervous tissue, yields the MR signal observable on the voxel scale (von dem Hagen and Henkelman, 2002). Thus, two effects are interwoven in the macroscopic diffusion signal, that is, the microscopic diffusion process and the neurite orientation distribution. Deconvolution techniques aim to recover the orientation distribution from diffusion MR measurements. These methods may be categorised according to the signal dictionary in which the estimation problem is solved. Examples are spherical harmonics (Tournier et al., 2004; Anderson, 2005), maximum-entropy regularisation (Alexander, 2005), mixtures of Bingham distributions (Kaden et al., 2007), reproducing kernel Hilbert spaces (Kaden et al., 2008; Kaden and Kruggel, 2011) and Dirichlet process mixtures (Kaden and Kruggel, 2012), among others. However, these dictionaries model only subsets of neurite orientation distributions, as the space of all orientation distributions is far too large to be computationally manageable, hence introducing approximation errors.

Another limitation of DTI and most spherical deconvolution methods is that they ignore the presence of multiple tissue components, such as neurons, their cellular extensions, neuroglia and extracellular space, that compartmentalise water and may have different signal properties. Multiple tensor models typically describe two or more microscopic compartments (Niendorf et al., 1996) but neglect axon crossings and orientation dispersion, or are interpreted as multiple fibre bundles (Tuch et al., 2002) but ignore compartmentalised water on the (sub-) cellular scale. Since there is no significant attenuation of the intra-axonal diffusion signal perpendicular to highly myelinated fibres (in the absence of microscopic undulation) at gradient strengths of |*G*|≤100 mT/m and echo times of *t*_TE_≤125 ms, it appears reasonable to set the transverse diffusivity inside the axons to zero, as done by Behrens et al. (2003a, 2003b, 2007). Their “ball-and-stick” model, however, considers only a discrete set of axon orientations, neglecting orientation dispersion within the fibre bundles. WMTI (Fieremans et al., 2011) infers various microstructural features in the case of a single axon orientation, which are calculated from metrics obtained with diffusion kurtosis imaging. Hereafter we shall ignore biophysical models (see Panagiotaki et al. (2012) for a review) that aim to recover structural parameters like the axon diameter because current clinical scanners (with |*G*|≤100 mT/m) lack sensitivity to such features (Drobnjak et al., 2016). Moreover, the assumption of a single axon orientation per voxel is grossly simplistic: even orientationally coherent white matter regions such as the corpus callosum exhibit significant directional heterogeneity featuring axon undulation and orientation dispersion (Axer et al., 2001; Mikula et al., 2012).

More recently, Jespersen et al. (2007, 2010) estimated the neurite density and per-dendrite/axon diffusion coefficients in the presence of complex orientation distributions using low-order spherical harmonics, which, however, exclude mixtures of Dirac masses and are impractical for crossings of three fibre bundles. These *post-mortem* studies of baboon and rat brain rest on rich MRI data sets with a multitude of *b*-values. The “ball-and-rackets” model (Sotiropoulos et al., 2012), a special case of parametric spherical deconvolution (Kaden et al., 2007), represents the directional tissue structure using mixtures of Bingham distributions. Their approach assumes that the parallel intra-axonal and isotropic extra-axonal diffusivities are equal within a voxel. The NODDI technique (Zhang et al., 2012) attempts to recover the neurite orientation dispersion and density. This method describes the axon orientation distribution with a single Watson distribution and thus does not account for fibre crossings, which can be found in large parts of the brain white matter (Schmahmann and Pandya, 2006). The technique also assumes a single and fixed intrinsic diffusivity for nervous tissue (in human *in-vivo* studies 1.7 μm^2^/ms) over the whole brain and across MRI protocols, subjects of different age and patients with different neurological conditions, which is doubtful and a major source of the systematic overestimation of free-water content in the cerebral white matter, in contrast to what is known from *T*_2_-relaxometry (MacKay et al., 1994) and neuroanatomy studies (Nieuwenhuys et al., 2008). Further, NODDI models the extra-neurite water pool in fast exchange over all neurite orientations, which is questionable since the microenvironment a diffusing water molecule covers during the observation time is orders of magnitudes smaller than the dimension of the voxel the measured signal comes from.

For the recovery of microscopic tissue features in the brain, it is clear that first and foremost we need to factor out the intra-voxel fibre orientation distribution. To achieve this, we use microscopic diffusion anisotropy mapping based on the Spherical Mean Technique (SMT). The key insight of the recently proposed method (Kaden et al., 2016) is that for any fixed gradient magnitude and timing, hence fixed *b*-value, the spherical mean of the diffusion signal over the gradient directions does not depend on the microdomain orientation distribution. In particular, the mean diffusion signal is only a function of the voxel-averaged microscopic diffusion process. This seminal result was formally proven for general microscopic diffusion models or, equivalently, impulse response functions. To demonstrate the approach, Kaden et al. (2016) have chosen, for low *b*-value measurements, a microscopic diffusion tensor that is rotationally symmetric, *i.e.* a second-order approximation of the microscopic diffusion process.

In this paper we extend SMT-based microscopic diffusion anisotropy imaging and introduce a multi-compartment model that takes the presence of multiple tissue components on the microscopic scale into account. The objective is to map, using clinically viable data, the neurite density and compartment-specific microscopic diffusivities unconfounded by the effects of fibre crossings and orientation dispersion. This multi-compartment microscopic model overcomes key limitations in existing techniques like WMTI (Fieremans et al., 2011) or NODDI (Zhang et al., 2012) as we make no assumptions about the neurite orientation distribution (*e.g.* single orientations, spherical harmonics or mixtures of Bingham distributions) and estimate the microscopic diffusion coefficients from the data. Once the microscopic diffusion signal has been uncovered, we reconstruct the fibre orientation distribution using spherical deconvolution, which, unlike traditional methods, utilises a spatially varying multi-compartment impulse response function, and calculate the orientation dispersion entropy to quantify directional tissue heterogeneity.

To demonstrate the developed technique, we use high-quality diffusion data acquired with a bespoke scanner system from the Human Connectome Project (Van Essen et al., 2012). The aim is to establish the normative values of the simple-to-estimate biomarkers for a large cohort of healthy young adults, which may then support clinical diagnostics in patients. Multi-compartment SMT can be immediately used in the clinic, as well as with various retrospective studies, since the method requires only a widely available off-the-shelf pulse sequence with two or more *b*-shells and uniformly distributed gradient directions runnable within clinically feasible scan times. Second, we showcase the potential of the new technique for the detection of pathological tissue alterations in a preclinical animal model of TSC, a genetic multi-organ disorder which also impacts the structural integrity of brain tissue and hence may lead to neurological manifestations such as autism, epilepsy and developmental delay (Crino et al., 2006). This *ex-vivo* MRI study uses conditional knockouts (CKO) of *Rictor* and *Tsc2* in *Olig2-Cre* mice, which both target the formation of oligodendrocyte precursors and oligodendrocyte differentiation (Carson et al., 2015; Kelm et al., 2016). A distinguishing feature in comparison to previous DTI-based studies (Jansen et al., 2003; Makki et al., 2007; Simao et al., 2010; Peters et al., 2012) is that the microscopic diffusion indices have factored out the neurite orientation distribution, which is useful for the evaluation of TSC pathology in tissue with complex directional architecture.

## Methods and materials

### Spherical Mean Technique

To examine microscopic tissue features unconfounded by the directional brain structure, we use the Spherical Mean Technique (SMT) to factor out the effects due to the neurite orientation distribution (Kaden et al., 2016). This method requires the parametric specification of a microscopic diffusion model describing the signal coming from a tissue microenvironment, which may be directionally anisotropic. The observable MR signal on the voxel scale is produced by a large population of microdomains that potentially have a complex orientation distribution. We assume that the microscopic tissue geometry is rotationally symmetric with rotation axis ***ω***∈*S*^2^, henceforth called orientation, where *S*^2^ = {*ω*∈ℝ^3^: ‖*ω*‖ =1} denotes the two-dimensional unit sphere. Let *b*≥0 denote the diffusion weighting factor and *g*∈*S*^2^ the normalised gradient direction, whilst keeping the timing of the pulse sequence fixed. Then the microscopic diffusion signal

(1) $$h_{b}(g,\omega )=h_{b}(\langle g,\omega \rangle )$$

depends only on the spherical distance 〈*g*,***ω***〉∈[−1,1] between any two points *g*,*ω*∈*S*^2^. We will use both notations in Eq. (1) interchangeably. The signal function *h_b_* is set to be antipodally symmetric, *i.e. h_b_*(*g*,***ω***)= *h_b_*(−*g*,***ω***).

Once the microscopic diffusion model has been defined, Kaden et al. (2016) proved that the spherical mean of the diffusion signal ${\bar{e}}_{b}$ over the gradient directions – with the other sequence parameters, in particular the gradient magnitude and timing, hence the diffusion weighting factor *b*, fixed – is invariant with respect to the neurite orientation distribution. More specifically, the mean diffusion signal takes the form

(2) $${\bar{e}}_{b}=\int_{0}^{\pi /2}h_{b}(\cos(\theta ))\sin(\theta )d\theta ,$$

where *θ* is an auxiliary variable which encodes the angle between the gradient direction and microdomain orientation. Eq. (2) shows that, for a given *b*-value, ${\bar{e}}_{b}$ is fully determined by the microscopic diffusion model. This insight has enabled us to estimate the tissue microanatomy unconfounded by and without knowledge of the directional brain architecture in a simple, fast and robust way. SMT exploits this invariance property using a two-step procedure as follows (Kaden et al., 2016). First, the spherical mean signal is computed by averaging the *T*_2-_normalised diffusion signals acquired with uniformly sampled gradient directions for each *b*-value separately. Second, the parameters of the microscopic diffusion model (1) are estimated using a least-squares technique that fits the spherical mean version (2) of the model to the measured mean signals for a set of diffusion weighting factors.

### Multi-compartment microscopic model

Next we shall develop a new microscopic diffusion model. The present work divides brain tissue into an intra-neurite domain and extra-neurite compartment. The former component consists of dendrites and axons, which may be surrounded by myelin sheath. A characteristic feature of these cellular extensions is their cylindrical geometry. The latter compartment includes neurons, glial cells, *e.g.* oligodendrocytes, neurolemmocytes and astrocytes, and extracellular space. The objective is to decompose microscopic diffusion anisotropy into signal components coming from the water pools inside and outside the neurites, respectively, based on the fact that these signal contributions are markedly different. Hence, the diffusion signal for a microscopic environment of brain tissue with orientation *ω*∈*S*^2^ is modelled as

(3) $$h_{b}(g,\omega )=\underset{\text{intra}-\text{neurite}}{\underset{︸}{v_{\mathrm{int}}h_{b}^{\mathrm{int}}(g,\omega )}}+\underset{\text{extra}-\text{neurite}}{\underset{︸}{\left(1-v_{\mathrm{int}})h_{b}^{\text{ext}}(g,\omega \right)}},$$

where $h_{b}^{\mathrm{int}}$ denotes the signal from the intra-neurite water pool, $h_{b}^{\text{ext}}$ the signal component due to the extra-neurite compartment and *v*_int_∈[0,1] the intra-neurite volume fraction. Since water between the myelin layers, because of its rapid *T*_2_-relaxation (MacKay et al., 1994), does not significantly contribute to the measured signal at sufficiently long echo times, we do not include a myelin compartment, but assume that the intra- and extra-neurite water pools have a similar *T*_2_-relaxation behaviour.

Under the assumption that the intra-neurite water pool is isolated from its surroundings, for low *b*-value measurements as commonly obtained in clinical practice the applied gradients are not strong and/or long enough to produce detectable attenuation of the signal component perpendicular to the neurites since the diameter of the dendrites and axons is too small. Therefore, we set the transverse microscopic diffusivity to zero as proposed in the “ball-and-stick” model (Behrens et al., 2003a, 2003b). The microscopic signal from the intra-neurite compartment reads

(4) $$h_{b}^{\mathrm{int}}(g,\omega )=\exp\left(-b{\langle g,\omega \rangle }^{2}\lambda \right),$$

where 0≤*λ*≤*λ*_free_ is the intrinsic diffusion coefficient parallel to the neurites and the upper bound *λ*_free_ is given by the free-water diffusivity. Note that *λ* is an apparent (or effective) parameter that depends not only on the diffusion process in the underlying material but also on the MRI experiment, such as the temporal profile of the gradient sequence (Grebenkov, 2010).

Furthermore, we take into account that the extra-neurite tissue compartment potentially features a directionally anisotropic geometry on the micrometre scale. Thus, it is reasonable to describe this signal component with a rotationally symmetric microscopic tensor model

(5) $$h_{b}^{\text{ext}}(g,\omega )=\underset{\text{longitudinal}}{\underset{︸}{\exp\left(-b{\langle g,\omega \rangle }^{2}\lambda \right)}}\underset{\text{transverse}}{\underset{︸}{\exp\left(-b\left(1-{\langle g,\omega \rangle }^{2}\right)\lambda_{⊥}^{\text{ext}}\right)}}.$$

The first term on the right-hand side describes the microscopic diffusion process in the surroundings parallel to the neurites, while the second term quantifies the microscopic diffusivity in the characteristic vicinity perpendicular to the axons and dendrites. The transverse extra-neurite diffusion coefficient is modelled as a function of the intra-neurite volume fraction *v*_int_ and intrinsic diffusivity *λ*. Here we use a basic approach to describing the microscopic diffusion process around the neurites, that is, the first-order tortuosity approximation $\lambda_{⊥}^{\text{ext}}=(1-v_{\mathrm{int}})\lambda$ which was derived for a system of randomly placed parallel cylinders of variable diameter with impermeable boundaries in the long-time diffusion limit using effective medium theory (Bruggeman, 1935; Sen et al., 1981; Szafer et al., 1995). Unlike NODDI (Zhang et al., 2012), SMT does not assume that there is a fast mixing of extraneurite water across all axon and dendrite orientations. The microenvironment to which the diffusion process is sensitive (on the scale of the root-mean-square displacement of the water molecules) includes only an infinitesimal fraction of the large ensemble of neurites inside the voxel. In addition, this assumption gives rise to certain abnormalities, which are discussed in Appendix A.

### Mean diffusion-signal model

Following (Kaden et al., 2016), we calculate the spherical mean of the diffusion signal for a large population of microdomains with potentially complex orientation distribution using Eq. (2). The mean diffusion signal takes the form

(6) $${\bar{e}}_{b}=\int_{0}^{\pi /2}h_{b}(\cos(\theta ))\sin(\theta )d\theta ,$$

where

(7) $${\bar{e}}_{b}^{\mathrm{int}}=\frac{\sqrt{\pi }\text{erf}\left(\sqrt{b\lambda }\right)}{2\sqrt{b\lambda }}$$

and

(8) $${\bar{e}}_{b}^{\text{ext}}=\exp(-b\lambda_{⊥}^{\text{ext}})\frac{\sqrt{\pi }\text{erf}\left(\sqrt{b[\lambda -\lambda_{⊥}^{\text{ext}}]}\right)}{2\sqrt{b[\lambda -\lambda_{⊥}^{\text{ext}}]}}$$

are the spherical mean signals from the intra- and extra-neurite water pools, respectively. *b*≥0 denotes the diffusion weighting factor and erf the error function. Then we fit the spherical mean version of the multi-compartment microscopic diffusion model provided in Eqs. (6) to (8), which have factored out the effects due to fibre crossings and orientation dispersion, to the measured mean signals for a set of *b*-values. Given an extra-neurite diffusion model $\lambda_{⊥}^{\text{ext}}$, the parameters to be estimated are the intra-neurite volume fraction *v*_int_∈[0,1] and intrinsic diffusion coefficient *λ* subject to the constraint 0≤*λ*≤*λ*_free_, where the free-water diffusivity *λ*_free_ (Mills, 1973) is circa 1.88 μm^2^/ms at 17 °C (which is used for the *ex-vivo* mouse study) and about 3.05 μm^2^/ms at 37 °C (for *in-vivo* human imaging). Thus, the recovered model parameters are ensured to lie within a physically meaningful range.

### Human data

To establish the normative values of the novel microscopic diffusion indices, we use high-quality data kindly provided by the Human Connectome Project, WU-Minn Consortium (500 Subjects Data Release, Washington University, June 2014, available online at http://www.humanconnectome.org). The data sets were acquired on a bespoke Siemens 3T MRI scanner equipped with a customised gradient insert featuring a maximum gradient strength of 100 mT/m (Van Essen et al., 2012). A Stejskal–Tanner sequence measured 90 uniformly distributed gradient directions for each *b*-shell of nominally 1000, 2000 and 3000 s/mm^2^, keeping the gradient timing fixed with pulse duration of 10.6 ms and pulse separation of 43.1 ms. Only the magnitude, hence the *b*-value, and the directions of the diffusion encoding gradients were altered during the experiment. Additionally, 18 images without diffusion weighting were acquired. The spin-echo EPI scan with echo time of 89.5 ms and repetition time of 5.52 s was performed using a multi-band sequence (Setsompop et al., 2012) with slice acceleration factor of 3. The diffusion data were acquired with in-plane phase encoding in both right-to-left and left-to-right directions. SENSE1 multiple-coil combination was applied (Roemer et al., 1990). The diffusion-weighted images with an isotropic voxel resolution of 1.25 mm covered the whole brain.

The data sets analysed in this study came from 100 unrelated adult subjects (47 male, aged 29.1±3.7 years). The magnitude images were preprocessed using HCP’s Minimal Preprocessing Pipeline, version 3.1 (Glasser et al., 2013). Briefly, the signal intensity was normalised across the scan, the susceptibility-induced distortions were eliminated using the two images acquired with reversed phase-encoding polarities, and the data sets were corrected for eddy-current artefacts and subject motion (Andersson and Sotiropoulos, 2016). Spatial distortions due to gradient field nonlinearities were rectified and the diffusion-weighting gradients were adjusted at each voxel. Finally, the diffusion images were aligned to the axes of MNI152 space using rigid transformations without scaling (Jenkinson et al., 2002; Greve and Fischl, 2009). We analyse the diffusion data in the native volume space, which is consistently oriented across the cohort and faithfully represents the subject’s brain size and shape. Since the MR signal was combined with SENSE1 from multiple receive coils, the noise regime of the magnitude signal is well described by a Rician distribution (Gudbjartsson and Patz, 1995), albeit data preprocessing may alter its characteristics to a certain extent. To minimise potential effects of the noise-induced bias, the measurements were adjusted accordingly (Kaden et al., 2016).

### Animal study

To test multi-compartment microscopic diffusion imaging for the evaluation of tuberous sclerosis neuropathology, we conducted an *ex-vivo* study with two knockout mouse models of TSC. The disease, inherited in an autosomal dominant manner or appearing sporadically due to spontaneous mutations, results from an inactivating mutation in either the *Tsc1* or *Tsc2* gene encoding hamartin and tuberin, respectively. These proteins control the activity of the mammalian target of rapamycin (mTOR) kinase, which regulates cell size, proliferation and differentiation (Tee et al., 2002). As a follow-on study to Carson et al. (2012, 2013), we used conditional knockouts of *Rictor*, the rapamycin-insensitive component of mTOR, and *Tsc2* in *Olig2-Cre* mice, which have been introduced recently (Carson et al., 2015; Kelm et al., 2016). Both models are expected to impact myelination in the central nervous system, as *Olig2* plays an important role in the formation of oligodendrocyte precursors and oligodendrocyte differentiation (Lu et al., 2000; Zhou et al., 2000). *Rictor*-deficient mice showed moderate adverse effects, whilst *Tsc2* CKO resulted in a phenotype with severe adverse effects, yet the mice from both models were able to live into adulthood. All animal procedures were completed with approval of the Vanderbilt University Institutional Animal Care and Use Committee.

Eight normal, five *Rictor* and five *Tsc2* P60 mouse brains were scanned on a 15.2 T Bruker Biospec system with a 35 mm quadrature volume coil at bore temperature of 17±0.5 °C (Kelm et al., 2016). The preparation of the excised brains followed standard procedures. In addition, the mouse brains were doped with 1 mM Gd-DTPA, lowering the *T*_1_-relaxation time to approximately 400 ms and thus increasing the SNR efficiency of the MRI scan. Note that D’Arceuil et al. (2007) found little change in various metrics obtained from diffusion tensor imaging over a wide range of Gd-DTPA concentrations of up to 10 mM. The data were acquired using a 3D diffusion-weighted fast spin-echo sequence with repetition time of 200 ms, echo time of 19.0 ms, echo spacing of 7.1 ms and echo train length of 4. The scan with 128 × 96 × 72 image matrix and 19.2×14.4×10.8 mm^3^ field of view covered the whole brain, resulting in an isotropic voxel resolution of 150 μm. Diffusion weighting was achieved with a Stejskal–Tanner experiment consisting of two *b*-shells of nominally 3000 and 6000 s/mm^2^ with 30 gradient directions each, which were uniformly distributed (Jones et al., 1999) and measured twice with the gradient polarity reversed. The timing of the gradients was fixed with pulse duration of 5 ms and pulse separation of 12 ms so that the same diffusion propagator is observed during the experiment. In addition, 5 images without diffusion weighting were collected. The total scan time was about 12 h per mouse brain.

The MR images were reconstructed from the *k*-space data using a custom preprocessing pipeline, which includes Gibbs ringing suppression via Hann windowing, 3D Fourier transform and magnitude computation. Further, the diffusion scans were corrected for cross-term effects using the images obtained with opposite gradient polarities (Neeman et al., 1991). The microscopic diffusion indices are estimated in native measurement space, thereby neglecting the nature of Rician noise because SNR in tissue is about 100 for the zero *b*-value images. For comparison of the *Rictor*- and *Tsc2*-deficient models with control mice, we arbitrarily chose a mouse brain from the control group as reference, to which all other subjects were transformed using the FNIRT tool for nonlinear registration, as implemented in FSL (2012). After spatial normalisation of the microscopic diffusion maps, the voxelwise significance of any population differences were evaluated between *Rictor* and control as well as *Tsc2* and normal mice via unpaired two-sample *t*-tests. We calculated the threshold-free cluster enhanced (TFCE) pseudo *t*-statistics using standard parameters (Smith and Nichols, 2009). Multiple comparison correction across space was carried out by exhaustive permutation testing (Winkler et al., 2014) subject to the masked brain, which then yielded family-wise error (FWE) corrected *p*-value maps.

After MRI scanning, three *Rictor*, four *Tsc2* and six normal mouse brains were sectioned for histological analysis. Following a midsagittal cut, we examined four white matter regions, *i.e.* the genu (GCC), midbody (MidCC) and splenium (SCC) of the corpus callosum as well as the anterior commissure (AC). The tissue sections were stained with 1% toluidine blue and scanned with an FEI Tecnai T12 transmission electron microscope at various magnifications. Since two sections from the *Tsc2* model were corrupted during processing, we added a fourth *Tsc2* subject. The images were segmented into myelin and non-myelin pixels (Otsu, 1979), from which we obtained histological measurements of myelin fraction *f*_myel_ and myelinated axon fraction *f*_ax_. The myelinated axon density *ρ*_ax_ was quantified by manually counting myelinated axons within the field of view. We refer the reader to Kelm et al. (2016) for technical details.

## Results

### Multi-compartment microscopic diffusion

**Fig. 1:**
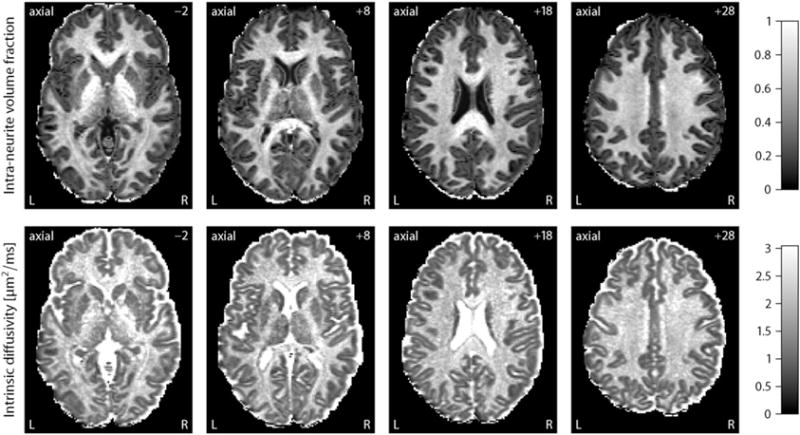
This plot depicts the intra-neurite volume fraction *v*_int_ (top) and intrinsic diffusivity *λ* for an example subject, shown for various slices in the axial plane from left to right. The key feature of these maps is that the confounding effects due to fibre crossings and orientation dispersion have been factored out. Abbreviations: left (L), right (R).

From a population of 100 unrelated young adults we have chosen a representative subject to demonstrate multi-compartment microscopic diffusion imaging. Fig. 1 maps the intra-neurite volume fraction *v*_int_ (top) and apparent intrinsic diffusivity *λ* for various slices in the axial plane. In comparison to previous work, we have factored out the neurite orientation distribution, including fibre crossings and orientation dispersion, to obtain these microscopic diffusion parameters. The figure shows that the neurite density index is markedly higher in brain white matter than in grey matter and microenvironments of white matter are heterogeneous across the cerebral white matter, even after the directional tissue structure has been integrated out. For instance, the intra-neurite volume fraction is higher in the corpus callosum and internal capsule compared to other white matter regions, which is presumably due to the converging pattern of the callosal fibres and corticospinal tract, respectively, that results in a reduction of the extra-neurite space together with a higher neurite density. Note that the intra-neurite volume fraction is measured with respect to the voxel volume excluding the myelin compartment because at an echo time of 89.5 ms the MR signal from myelin water is almost fully attenuated due to its short *T*_2_-relaxation time. Further, our data analysis suggests that the intrinsic diffusivity, which is the microscopic diffusion coefficient parallel to the neurites, varies substantially over the brain and is on average significantly higher in white matter tissue than earlier assumed (Zhang et al., 2012). The estimation of this parameter from the data is a key advantage of the new technique, as the intrinsic diffusivity strongly influences the quantification of other structural features such as the neurite density.

**Fig. 2:**
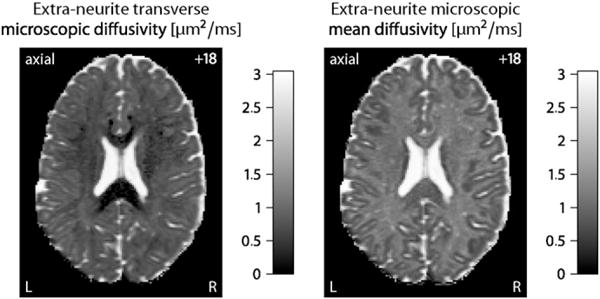
The left-hand side maps the transverse microscopic diffusivity of the extra-neurite water pool, while the extra-neurite microscopic mean diffusivity is shown on the right-hand side. Both indices provide useful contrast mechanisms for cerebrospinal fluid. The number in the upper right corner denotes the plane in unscaled MNI152 space.

Another noteworthy result is that in the ventricular system and subarachnoid space the intrinsic diffusivity approaches the diffusion coefficient of free water and the intra-neurite volume fraction, *i.e.* the signal component of highly anisotropic microscopic diffusion, tends to zero. Fig. 2 (left) maps the transverse microscopic diffusivity $\lambda_{⊥}^{\text{ext}}$ outside the neurites as a function of the intra-neurite volume fraction *v*_int_ and intrinsic diffusivity *λ*, here $\lambda_{⊥}^{\text{ext}}=(1-v_{\mathrm{int}})\lambda$. The right plot of this figure shows the microscopic mean diffusivity of the extra-neurite water pool, which is defined as

(9) $$\begin{array}{l} {\bar{\lambda }}^{\text{ext}}=\frac{1}{3}(\lambda_{\|}^{\text{ext}}+2\lambda_{⊥}^{\text{ext}})\\ \;=\left(1-\frac{2}{3}v_{\mathrm{int}}\right)\lambda , \end{array}$$

where $\lambda_{\|}^{\text{ext}}$ denotes the extra-neurite longitudinal microscopic diffusivity which equals *λ*. The figure demonstrates that the estimated microscopic diffusivities of the extra-neurite compartment are close to the self-diffusion coefficient of free water in the ventricles and around the cerebral cortex. It is straightforward to segment regions with high free-water content from these maps; especially the extra-neurite microscopic mean diffusivity ${\bar{\lambda }}^{\text{ext}}$ is a useful biomarker of cerebrospinal fluid, whose microscopic diffusion process is isotropic. In summary, the intra-neurite volume fraction and intrinsic diffusivity provide contrast between different types of nervous tissue without the confounding effects of fibre crossings and orientation dispersion. Multi-compartment microscopic diffusion imaging is also able to discriminate cerebrospinal fluid, even though the underlying model does not have a dedicated free-water component. Alternatively, we may eliminate partial volume effects due to cerebrospinal fluid contamination by adding a fluid attenuated inversion recovery (FLAIR) sequence.

### Sparse gradient sampling

**Fig. 3:**
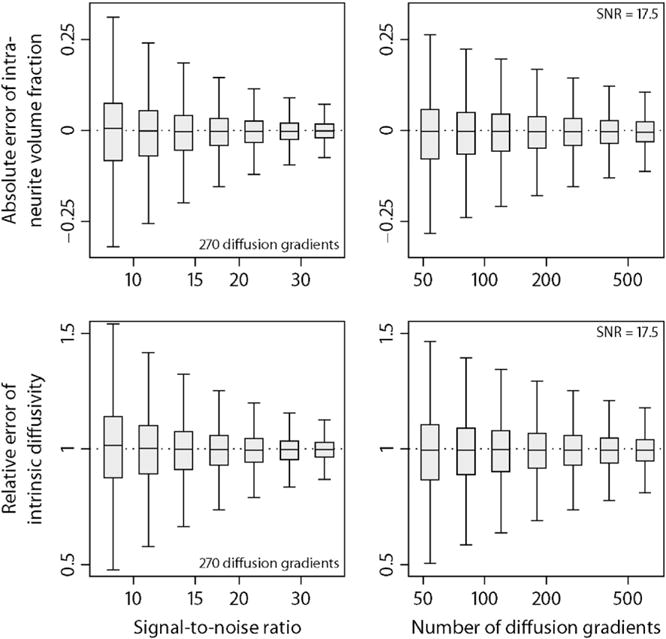
Estimation accuracy of the intra-neurite volume fraction (top) and intrinsic diffusivity. In the left column the estimation errors are shown for the acquisition protocol and various signal-to-noise ratios. The right column depicts box-and-whisker plots (with 1.5 times the interquartile range) for different numbers of diffusion gradients evenly distributed over three *b*-shells. See text for more details about the simulation study.

To demonstrate the reliability of SMT, we simulate fibre orientation distributions that closely resemble the tissue geometry of white matter. Here a Dirichlet process mixture with bipolar Watson kernel (Kaden and Kruggel, 2012; Kaden et al., 2016) is used to draw random spherical density functions, which include a broad range of fibre crossings and orientation dispersion. In fact, under the topology of weak convergence the Dirichlet process mixture includes all orientation distributions in its closure. The spherical convolution of these synthetic distributions with the multi-compartment impulse response function (3) yields the diffusion signals, which are then disturbed by Rician noise. For our simulation experiments the ground-truth intra-neurite volume fraction *v*_int_ and intrinsic diffusivity *λ* are uniformly drawn from the intervals [0,1] and [0,*λ*_free_], respectively. 50,000 trials each were run to investigate the estimation error of the microscopic diffusion indices under various scenarios after adjustment for the Rician noise bias. Fig. 3 shows the absolute error of the intra-neurite volume fraction and the relative error of the intrinsic diffusivity as a function of the signal-to-noise ratio (SNR, left column), using the human acquisition protocol, and of the total number of diffusion gradients evenly distributed over three *b*-shells, here 1000, 2000 and 3000 s/mm^2^. The fixed parameter is indicated in a corner of the plots. The box-and-whisker diagrams demonstrate that the variance of the estimator decreases as the signal-to-noise ratio and/or the number of diffusion gradients increase. This average-case study over density functions drawn from a Dirichlet process mixture suggests that SMT is a robust estimator of *v*_int_ and *λ*. The simulations also show that adverse effects due to the Rician noise regime are removed to a large extent.

**Fig. 4:**
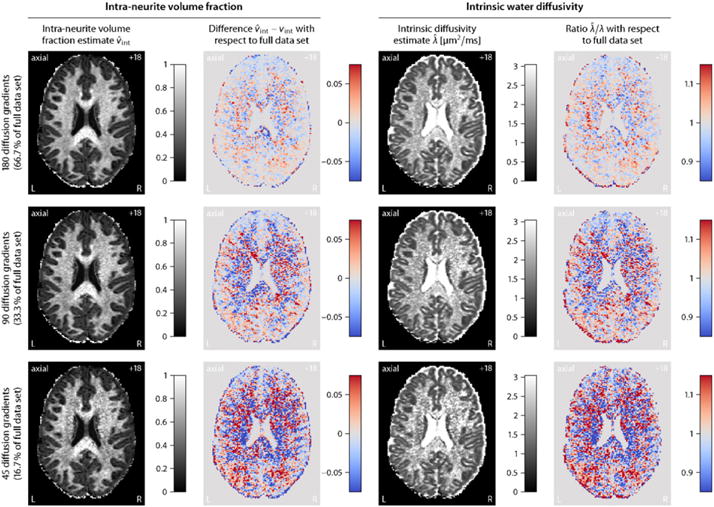
Sparse gradient sampling for (from top to bottom) 66.7%, 33.3% and 16.7% subsets of diffusion gradients evenly distributed over three *b*-shells, here 1000, 2000 and 3000 s/mm^2^. The first two columns depict the intra-neurite volume fraction *v*_int_ and difference ${\hat{v}}_{\mathrm{int}}-v_{\mathrm{int}}$, while in the last two columns the intrinsic diffusivity *λ* and ratio $\hat{\lambda }/\lambda$ are shown. The reference neurite density index *v*_int_ and intrinsic water diffusivity *λ* are both estimated from the full data set (cf. Fig. 1).

The HCP data sets were acquired on a bespoke scanner system using a sophisticated imaging protocol with lengthy scan time, which leads to unique data sets with unprecedented quality but extends only partially to clinical practice that generally needs to cope with modest hardware and limited time for MRI examination. These high-quality data total 270 diffusion-encoding gradients evenly distributed over three *b*-shells. Since the imaging gradients and field nonlinearities give rise to small spatial variations in the diffusion weighting factor, we will use the nominal *b*-values, in the present study 1000, 2000 and 3000 s/mm^2^, to refer to them. The following experiment subsamples the diffusion gradients, noting that those gradients were arranged in a way so that a subset of the first *n* directions, together with their antipodal points, are approximately evenly distributed on the sphere (Caruyer et al., 2013). Fig. 4 plots the intra-neurite volume fraction *v*_int_ and intrinsic diffusivity *λ* for subsets of 180, 90 and 45 diffusion gradients over the three *b*-shells. This figure also maps the difference ${\hat{v}}_{\mathrm{int}}-v_{\mathrm{int}}$ and ratio $\hat{\lambda }/\lambda$ with respect to *v*_int_ and *λ*, which were estimated from the full data set, respectively. Fig. 4 demonstrates that SMT-based multi-compartment microscopic diffusion imaging produces reliable results with much less diffusion-sensitising gradients within clinically feasible scan times, but at the expense of a noisier appearance of the microscopic diffusion indices. Note, however, that these difference/ratio maps overestimate the estimation error since the microscopic diffusion parameters fitted from all 270 diffusion gradients are also subject to errors.

**Fig. 5:**
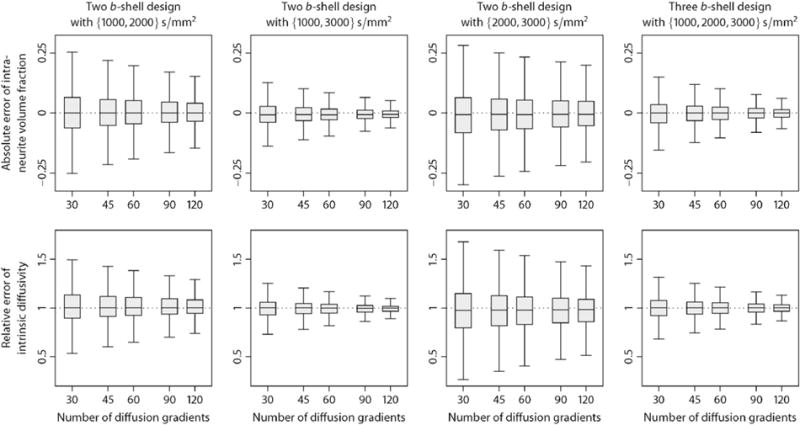
Random subsampling analysis for determining the estimation precision of the intra-neurite volume fraction (top) and intrinsic diffusivity as a function of the total number of diffusion gradients for different *b*-shell designs. The box-and-whisker plots (with 1.5-times the interquartile range) quantify the absolute and relative errors, which are calculated with respect to the estimates obtained from the full data set.

Next we sampled random subsets of diffusion gradients from the full data set (without replacement) to study the estimation precision of the new imaging biomarkers quantitatively. SMT requires two or more *b*-shells for the recovery of the intra-neurite volume fraction and intrinsic diffusivity because otherwise the estimation problem is underdetermined. In detail, for the representative subject we investigated four different *b*-shell designs with {1000, 2000}, {1000, 3000}, {2000, 3000} and {1000, 2000, 3000} s/mm^2^ over which various numbers of diffusion gradients were evenly distributed. Fig. 5 shows the results of this random subsampling analysis, which was repeated 100-times for each gradient design configuration. The top row of this figure plots the absolute estimation error of the intra-neurite volume fraction, while in the bottom row the relative error of the intrinsic diffusivity is shown, both with respect to the full data set. The estimation error decreases with an increasing number of diffusion gradients. Moreover, a two-shell design with {1000, 3000} s/mm^2^ is statistically more efficient than two-shell designs with {1000, 2000}, {2000, 3000} s/mm^2^ or a three-shell design with {1000, 2000, 3000} s/mm^2^, which suggests that the *b*-values should be separated from each other for better performance. In conclusion, we have demonstrated the clinical applicability of the developed imaging technique, which is able to recover microscopic diffusion-based features without the confounding effects due to the neurite orientation distribution.

### Normative database

**Fig. 6:**
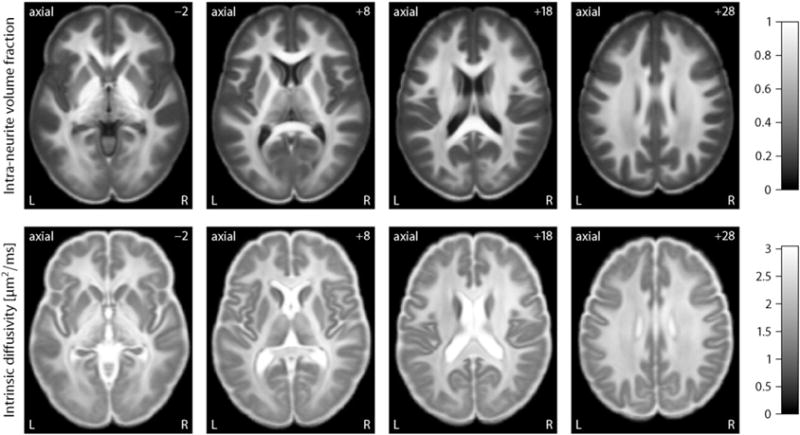
This plot displays the population mean of the intra-neurite volume fraction *v*_int_ (top) and intrinsic diffusivity *λ* over 100 unrelated subjects, shown for various slices in the axial plane from left to right. These multi-compartment microscopic diffusion maps establish the normative values of the novel biomarkers for a cohort of healthy young adults.

**Fig. 7:**
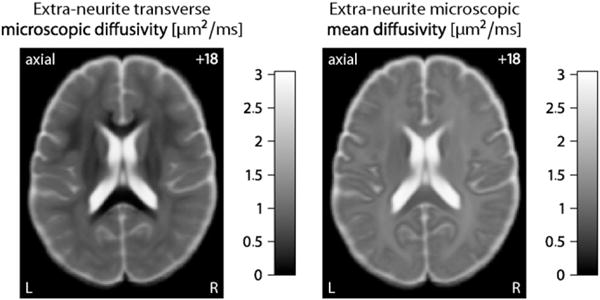
The left-hand side plots the extra-neurite transverse microscopic diffusivity, while the microscopic mean diffusivity of the extra-neurite water pool is shown on the right-hand side. Both parameter maps are voxel-wise averages over a cohort of 100 healthy young adults. The number in the upper right corner denotes the plane in MNI152 space.

Next we establish the normative values of the new microscopic diffusion indices over a population of 100 healthy young adults coming from the HCP data set. For this purpose we spatially normalise the parameter maps, which were obtained in native measurement space, across the subjects using HCP’s Minimal Preprocessing Pipeline, version 3.1 (Glasser et al., 2013). Briefly, the estimated maps were transformed into MNI152 space using the FNIRT tool for non-linear registration so that the brain size and shape are the same for all participants. Fig. 6 depicts the voxel-wise population averages of the intra-neurite volume fraction *v*_int_ (top) and intrinsic diffusivity *λ*, which are shown for various axial slices. Note that the number in the upper right corner denotes here the plane in standard MNI152 space. All observations previously made on the representative subject extend to the cohort of 100 unrelated adults, which demonstrates the consistency and reproducibility of the developed method. For instance, the neurite density index is considerably higher in brain white matter than in grey matter and the intra-neurite volume fraction is increased in the corpus callosum and internal capsule compared to other white matter regions. Fig. 7 maps the transverse microscopic diffusivity (left) and microscopic mean diffusivity of the extra-neurite water pool averaged across the subjects in stereotactic coordinate space. We observe that in the ventricular system and sub-arachnoid space the extra-neurite microscopic mean diffusivity approaches the self-diffusion coefficient of free water.

**Fig. 8:**
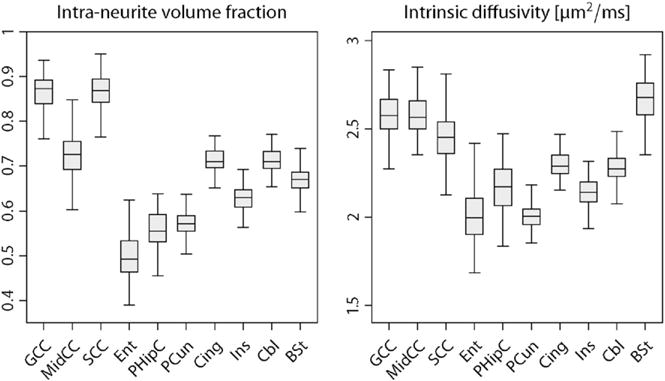
Box-and-whisker plots (with 1.5-times the interquartile range) of the intra-neurite volume fraction *v*_int_ (left) and intrinsic diffusivity *λ* in various white matter regions for a population of 100 unrelated subjects. Abbreviations: genu (GCC), midbody (MidCC) and splenium (SCC) of the corpus callosum, entorhinal (Ent), parahippocampal (PHipC), precuneus (PCun), cingulate (Cing), insula (Ins) and cerebellum (Cbl) white matter, brain stem (BSt).

For a region-based population analysis the brain white matter was automatically segmented using HCP’s Minimal Preprocessing Pipeline, where the cortical parcellation obtained by FreeSurfer (2014) was expanded to white matter. A distance constraint halted the label propagation after 5 mm, thus producing an anatomical parcellation of the gyral white matter (Salat et al., 2009). Subsequently we computed the mean of the parameter under consideration over a region of interest in native measurement space for each subject. Fig. 8 summarises the empirical distribution of the intra-neurite volume fraction *v*_int_ and intrinsic diffusivity *λ* in various white matter regions for a cohort of 100 healthy young adults using box-and-whisker plots (with 1.5-times the interquartile range). The figure shows that the neurite density index, which is obtained with respect to the voxel volume excluding the myelin compartment since the echo time of 89.5 ms is significantly longer than the *T*_2_-relaxation time of myelin water, is substantially higher in the corpus callosum, which is most likely due to the dense packing of the converging fibres. The plot further illustrates the microscopic diffusion-based metrics in entorhinal, parahippocampal and precuneus white matter, which were recently proposed to be important for understanding entorhinal cortex pathophysiology and its propagation over the brain in early preclinical stages of Alzheimer’s disease (Khan et al., 2014). A key feature of the developed biomarkers is that they do not depend on the directional tissue architecture. Moreover, it is evident that the intrinsic diffusivity is not invariant in white matter and significantly higher than previously suggested (Zhang et al., 2012).

### Neurite orientation distribution

**Fig. 9:**
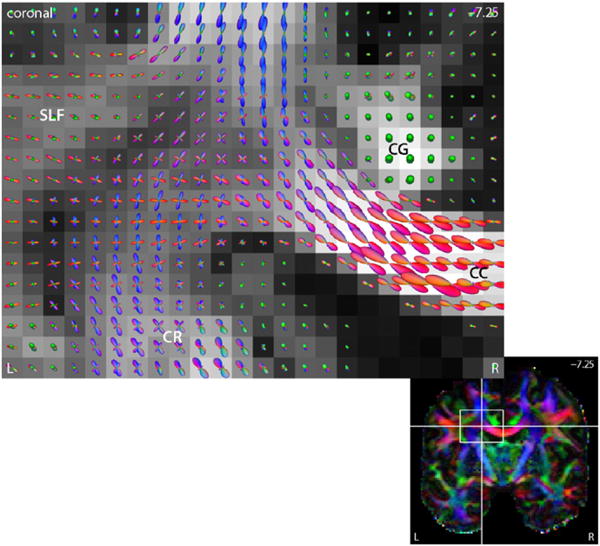
The plot depicts the neurite orientation distribution estimated by a reproducing kernel Hilbert space (RKHS) technique (Kaden et al., 2008; Kaden and Kruggel, 2011). The fibre orientation field reveals the radiation of the corpus callosum (CC), the corona radiata (CR) and their crossing, shown in the coronal plane. The underlying map displays the standard fractional anisotropy. Abbreviations: cingulum (CG), superior longitudinal fasciculus (SLF).

Once the microscopic diffusion process has been recovered voxel by voxel, we are able to estimate the fibre orientation distribution from the diffusion measurements using spherical deconvolution (Tournier et al., 2004; Anderson, 2005). Knowledge of the microscopic diffusivities is crucial for the specification of the impulse response function and hence the quantitative estimation of neurite orientation dispersion. The density function may be found in a reproducing kernel Hilbert space (Kaden et al., 2008; Kaden and Kruggel, 2011), which does not truncate the spherical harmonic expansion and ensures its characteristic properties, namely antipodal symmetry, non-negativity and normalisation with one. In previous work a microscopic diffusion tensor was used as impulse response function, which is here replaced by the multi-compartment model formulated in Eqs. (3) to (5). Fig. 9 demonstrates the recovery of the neurite orientation distribution from all *b*-shell data for a representative subject. The density function *p* is visualised by the quasi-spherical surface *S*^2^∋***ω***↦*p*(***ω***)***ω***∈ℝ^3^. The figure shows the crossing of the callosal fibres and corona radiata in the centrum semiovale of the left hemisphere. Further, the orientation dispersion in the corpus callosum seems to be higher than previous studies have suggested, but is in agreement with histological findings (Axer et al., 2001; Mikula et al., 2012). We may also use alternative deconvolution techniques based on, for example, mixtures of Bingham distributions (Kaden et al., 2007; Kaden and Kruggel, 2012).

**Fig. 10:**
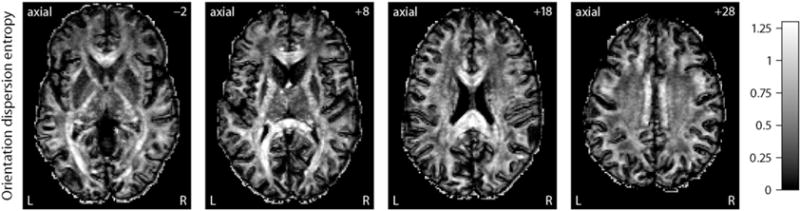
These scalar maps depict a summary statistics of the neurite orientation distribution in native measurement space, shown for various slices in the axial plane from left to right. The orientation dispersion entropy is defined as Kullback–Leibler divergence (or relative entropy) of the fibre orientation distribution with respect to the uniform spherical distribution as reference measure.

Moreover, we may calculate summary statistics of the neurite orientation distributions. A useful example is the relative entropy *H*(*p*) of the density function *p* : *S*^2^ → [0, ∞), which is defined as Kullback–Leibler divergence

(10) $$\begin{array}{l} H(p)\;=D_{\text{KL}}(p,q)\\ \;=\int_{S^{2}}p(\omega )\ln\left(\frac{p(\omega )}{q(\omega )}\right)d\omega \end{array}$$

with respect to a reference measure, here the uniform distribution *q*(*ω*)=1/(4*π*), *ω*∈*S*^2^ on the sphere. Fig. 10 shows maps of the orientation dispersion entropy for various slices in the axial plane, which were estimated in native measurement space. In contrast to previous work, this index takes the full axon orientation distribution into account. The orientation dispersion entropy is close to zero when the estimated microdomain orientation distribution approaches the spherical uniform distribution, such as in the ventricular system, subarachnoid space and parts of the grey matter. In the corpus callosum, internal capsule and other white matter regions mainly formed by a single fibre bundle with a coherent orientational structure, the relative entropy of the neurite orientation distribution is high. Whereas the orientation dispersion entropy describes the directional tissue architecture, the fractional anisotropy of the classical tensor model encodes both the microscopic diffusion process and the neurite orientation distribution. We obtain similar results for the other subjects studied in this work. To conclude, SMT does not only allow to recover the microscopic diffusion process but also facilitates the quantitative estimation of neurite crossings and orientation dispersion, for the first time without any assumptions on unknown diffusivities.

### Tuberous Sclerosis Complex

**Fig. 11:**
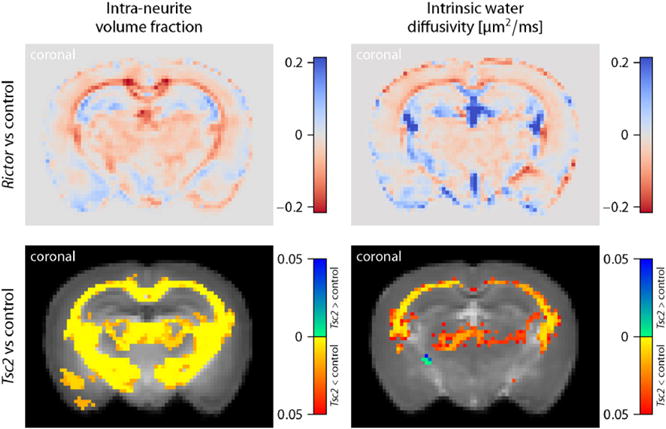
The plot shows the voxelwise difference of the population means between *Rictor*-deficient and control mice (top) as well as FWE-corrected *p*-value maps quantifying the significance of group differences between *Tsc2*-deficient and normal mice for the intra-neurite volume fraction *v*_int_ (right) and intrinsic diffusivity *λ*. The underlying maps in the bottom diagrams display the control group averages of the respective biomarkers.

**Fig. 12:**
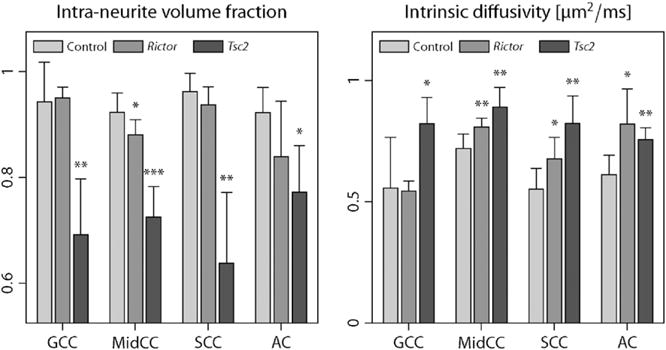
This region-based group analysis depicts the population mean and standard deviation of the intra-neurite volume fraction *v*_int_ (right) and intrinsic diffusivity *λ* for four white matter regions in the midsagittal plane. Significant group differences of *Rictor*- and *Tsc2*-deficient mice compared to normal controls are indicated with (*) *p*≤0.05, (**) *p*≤0.01 and (***) *p*≤0.001. Abbreviations: genu (GCC), midbody (MidCC), splenium (SCC) of the corpus callosum, anterior commissure (AC).

The following *ex-vivo* study of age-matched mice demonstrates that features of the microscopic diffusion process provide valuable biomarkers sensitive to TSC-induced abnormalities in the brain microstructure. Fig. 11 plots the voxelwise difference of the population means we observe between *Rictor* CKO and controls (top) as well as FWE-corrected *p*-value maps quantifying the significance of voxelwise group differences between *Tsc2* CKO and normal mice for the intra-neurite volume fraction *v*_int_ (left) and intrinsic diffusivity *λ*. The underlying maps in the bottom diagrams display, in the coronal plane, the population average of the microscopic diffusion indices over normal controls. Moreover, we study the four white matter regions examined in histology, *i.e.* the genu (GCC), midbody (MidCC) and splenium (SCC) of the corpus callosum as well as the anterior commissure (AC) in the midsagittal plane, which were delineated in the population average of the control mice. Fig. 12 shows a region-based group analysis for *Rictor* and *Tsc2* CKO with respect to normal controls using unpaired two-sample *t*-tests. For *Rictor*-deficient mice, which result in a phenotype with moderate adverse effects, we observe only minor deviations in the SMT estimates (which are not statistically significant in the voxel-level analysis), whereas *Tsc2*-deficient mice show a significant decrease in the neurite density index over wide areas of the brain white matter, presumably leading to the severe adverse effects seen in this TSC model. The abnormal intrinsic diffusivities suggest an altered intra- and/or extracellular milieu due to ongoing neuropathological processes. Further, in contrast to the *in-vivo* human data sets, we observe, at a *b*-value of 6000 s/mm^2^, a relatively high diffusion signal for gradient directions parallel to the main fibre orientation in the corpus callosum and other white matter regions, suggesting the presence of a signal component of very slow diffusion (Stanisz et al., 1997; Alexander et al., 2010), which, however, is not explicitly modelled here.

**Fig. 13:**
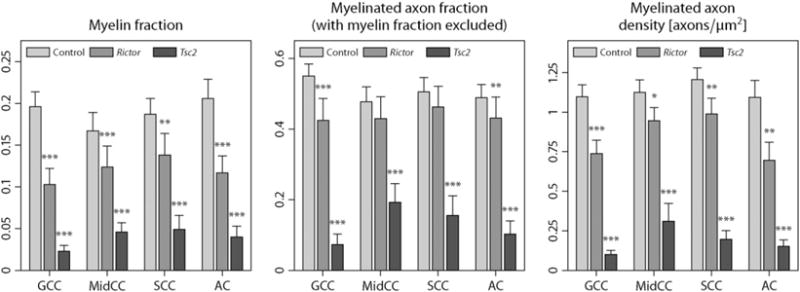
Statistical analysis of quantitative histology measures, showing the group mean and standard error of the myelin fraction *f*_myel_, myelinated axon fraction with the myelin fraction excluded *f*_ax_/(1−*f*_myel_) and myelinated axon density *ρ*_ax_ (from left to right) for control, *Rictor* and *Tsc2* CKO mice as well as the significance of group differences with respect to normal controls. Significance codes: (*) *p*≤0.05, (**) *p*≤0.01, (***) *p*≤0.001.

Finally, we report quantitative histology results from a subsample of the experimental animals. An initial examination shows that the overall architecture of the mouse brains remains intact, but with diffuse hypomyelination. In addition, decreased oligodendrocyte number was appreciated in the *Tsc2* CKO. As seen with other mouse models of TSC, we could not observe cerebral tubers, *i.e.* benign focal malformations at the grey-white matter junction disrupting the normal lamination of the cortex and a hallmark of the disease, nor a gross inflammatory response (Carson et al., 2015; Kelm et al., 2016). Fig. 13 shows (from left to right) the group mean and standard error of the histological myelin fraction *f*_myel_, myelinated axon fraction with the myelin fraction excluded *f*_ax_/(1−*f*_myel_), where *f*_ax_ denotes the histological axon fraction, and myelinated axon density *ρ*_ax_, *i.e.* the number of myelinated axons per unit area, for four white matter regions in normal controls, *Rictor*- and *Tsc2*-deficient mice. Significant group differences of the respective histological measures compared to controls, based on a Wilcoxon rank-sum test, are indicated. This figure demonstrates a reduction of the myelin fraction and myelinated axon density, thus hypomyelination, in the *Rictor* and especially *Tsc2* CKO. The decrease in myelinated axon fraction (with the myelin fraction excluded) is reflected by a reduction of the MRI-based intra-neurite volume fraction, which has excluded the myelin compartment because of its short *T*_2_-relaxation time, but generally includes unmyelinated axons. Note that observed abnormalities in the MRI measure may be not only due to alterations in the total axon density, but also secondary effects of reduced myelination, which might lead to substantial water exchange between the intra- and extra-neurite compartments during the diffusion time.

## Discussion

This paper has introduced multi-compartment microscopic diffusion anisotropy imaging based on the recently proposed Spherical Mean Technique (SMT, Kaden et al., 2016). The rationale of this technique is that the macroscopic signal we measure on the voxel scale conflates two physical effects, which are microscopic diffusion anisotropy and the microdomain orientation distribution. Both features are crucial to data analysis since, if the microscopic environments were uniformly oriented and/or not directionally anisotropic, we would not be able to observe macroscopic diffusion anisotropy. Because of that, a primary aim of modern diffusion MRI in neuroscience research and clinical neurology is to disentangle these two effects. SMT-based microscopic diffusion anisotropy mapping has enabled us to do this, using off-the-shelf sequences with two (or more) *b*-shells achievable on standard clinical scanners. Once the two key contributors of the diffusion signal have been separated from each other, we are able to recover microstructural parameters in the presence of directional heterogeneity. Examples are the neurite density index and intrinsic diffusivity in nervous tissue, as shown in Figs. 1 and 6. At the same time, we can quantify the microdomain orientation distribution, including fibre crossings and orientation dispersion that are ubiquitous in the brain, and calculate summary statistics such as the orientation dispersion entropy (compare Figs. 9 and 10).

### Model assumptions

To establish clinical practicability, we need to limit the complexity of the diffusion model and thus the amount of experimental data required to fit its parameters robustly, which inevitably means that we need to make simplifying assumptions. It may then come to a surprise that a distinguishing feature of SMT (Kaden et al., 2016) is that it makes no assumptions about the *a priori* unknown orientation distributions and hence recovers the microscopic features in an unbiased way. The technique is solely based on the insight that for any fixed gradient magnitude and timing, thus fixed *b*-value, the spherical mean of the diffusion signal over the gradient directions does not depend on the neurite orientation distribution, but is only a function of the microscopic diffusion process. In this paper we have proposed a new multi-compartment model decomposing the microscopic signal into intra- and extra-neurite water pools, which are described by two rotationally symmetric microscopic diffusion tensors. The developed model does not include a myelin compartment, as the *T*_2_-relaxation time of water between the myelin layers is much shorter than the echo time of standard clinical scans. Therefore, the obtained volume fractions should be interpreted accordingly.

We impose three constraints on the double-microscopic-tensor model since otherwise the estimation problem is underdetermined for two-shell diffusion data, which can be easily seen after factoring out the microdomain orientation distribution using SMT. Compare also Jelescu et al. (2016) for an exhaustive analysis of a related model. First, we assume that the effective transverse diffusivity inside the neurites is zero (Behrens et al., 2003a, 2003b). This is a sensible choice for myelinated axons because the myelin sheath isolates the intra-neurite water pool from the surroundings to a large extent and the low *b*-value measurements typically performed in clinical practice do not give rise to any significant attenuation due to the small diameter of the axons. However, for unmyelinated axons and dendrites this approximation may hold only partially because of the permeability of membranes and possible neurite undulation on the microscopic scale (Nilsson et al., 2012). Second, the proposed method does not differentiate between the longitudinal microscopic diffusivities inside and outside the axons and dendrites (which might be different), but estimates a voxel average over the intra- and extra-neurite compartments. A key feature of the present work is that the intrinsic water diffusivity, which describes the hindered diffusion process across neurons and glia as well as cell organelles and cytoskeleton, is obtained from the data. Our results in Figs. 1, 6, 8 and 11 demonstrate that this parameter varies markedly in the brain, reflecting the fine-structural variability of the underlying cellular milieu.

In contrast to previous work, we do not approximate the extra-neurite signal component with an isotropic diffusion model (Behrens et al., 2007; Jespersen et al., 2007; Sotiropoulos et al., 2012), but allow for microscopic anisotropy in the case of high neurite densities. As a third constraint, the transverse microscopic diffusion in the extra-neurite compartment is represented as a function of the intra-neurite volume fraction and intrinsic diffusivity using a first-order approximation of the tortuosity effect (Bruggeman, 1935; Sen et al., 1981; Szafer et al., 1995). This model is based on effective medium treatment of a system of randomly placed parallel cylinders of variable diameter with impermeable boundaries in the long-time diffusion limit, which may only partially reflect the underlying microgeometry because of microscopic neurite undulation and the permeability of membranes in unmyelinated axon and dendrites. Alternatively, we may use a more advanced extra-neurite model (Novikov and Fieremans, 2012; Novikov et al., 2014) or estimate the transverse microscopic diffusivity outside the neurites from the data, which, however, requires a more sophisticated experiment design. Especially in highly densely packed white matter regions like the corpus callosum, where the microscopic diffusivity perpendicular to the axons is very low, diffusion measurements with higher *b*-values may be able to resolve the transverse microscopic diffusion process more accurately. Although the general approach of SMT naturally extends to more complex microscopic diffusion models as formally proven by Kaden et al. (2016), the presented multi-compartment model provides simple-to-estimate markers of microstructural tissue features achievable on standard scanners, acknowledging the tight time constraints in clinical settings.

### Comparison with WMTI and NODDI

In the following we compare multi-compartment microscopic diffusion imaging with two related techniques. WMTI (Fieremans et al., 2011) attempts to infer microstructural parameters from metrics obtained with diffusion kurtosis imaging. This method provides independent estimates of the intra-axonal volume fraction *v*_int_, the intra-axonal longitudinal diffusivity $\lambda_{\|}^{\mathrm{int}}$, the extra-axonal longitudinal diffusivity $\lambda_{\|}^{\text{ext}}$ and the extra-axonal transverse diffusivity $\lambda_{⊥}^{\text{ext}}$, thereby setting the intra-axonal transverse diffusivity $\lambda_{⊥}^{\mathrm{int}}$ to zero and assuming that the tangential distribution of the intra-voxel fibre population at millimetre resolution is a Dirac mass, which means that all axons form straight lines and run parallel to each other. The latter is overly simplistic as fibre crossings and orientation dispersion are ubiquitous in the human brain. Even in the corpus callosum the directional architecture is far from homogeneous (Axer et al., 2001; Mikula et al., 2012). In contrast, SMT does not make any assumptions about the fibre orientation distribution and hence is universally applicable. The proposed technique estimates a voxel average of $\lambda_{\|}^{\mathrm{int}}$ and $\lambda_{\|}^{\text{ext}}$, which we call intrinsic diffusivity *λ*, and the extra-neurite transverse diffusivity is inferred from *λ* and the intra-neurite volume fraction *v*_int_, as demonstrated in Figs. 2 and 7. Furthermore, even if the diffusion signal from a single microdomain is modelled by a microscopic diffusion tensor (thus is mono-exponential in all directions), the diffusion signal observed at the voxel level is in general not mono-exponential for complex orientation distributions. Fieremans et al. (2011) made no attempt to relate higher-order effects seen in diffusion kurtosis imaging to directional tissue heterogeneity.

The NODDI technique (Zhang et al., 2012) models the neurite orientation distribution with a single Watson density and hence ignores fibre crossings which are a distinctive feature of human connectional neuroanatomy. Specifically, Kaden et al. (2007) showed in a diffusion MRI study that the majority of white matter voxels features multiple fibre bundles whose accurate representation requires two or more Bingham distributions. In comparison, SMT is free of orientation distribution models. NODDI assumes a single and fixed intrinsic diffusivity *λ* (in human *in-vivo* studies 1.7 μm^2^/ms), whereas the developed method estimates the microscopic diffusion coefficients from the data. Figs. 1, 6, 8 and 11 demonstrate that *λ* varies significantly over the brain white matter with an average value that is considerably higher than earlier assumed (Zhang et al., 2012). We also expect to see differences in *λ* across age and neurological conditions, making the intrinsic diffusivity a valuable biomarker (compare Figs. 11 and 12). Moreover, the underestimation of *λ* in NODDI gives rise to a systematic overestimation of free-water content in the cerebral white matter, which stands in contrast to *T*_2_-relaxometry (MacKay et al., 1994) and well-known neuroanatomy (Nieuwenhuys et al., 2008), and may adversely affect the recovery of other parameters such as their neurite density index. Lastly, the NODDI method assumes that the extra-neurite water pool is in fast exchange across all neurite orientations, which is doubtful and leads to contradictory results as detailed in Appendix A. Multi-compartment microscopic diffusion imaging overcomes these model inconsistencies.

### Tuberous Sclerosis Complex

Pathological manifestations of tuberous sclerosis are manifold, but the impairment of the structural integrity of brain tissue contributes substantially to the morbidity seen in patients. Recent studies with mouse models of TSC (Meikle et al., 2008; Carson et al., 2012, 2013) suggested that global diffuse changes in white matter might give rise to universal cortical dysfunction together with various neuropsychiatric conditions, in addition to the multifocal tuber pathology. Indeed, Marcotte et al. (2012) demonstrated widespread microstructural alterations distinct from tubers in human patients via histological analysis of *post-mortem* brain specimen. This *ex-vivo* MRI study with conditional knockouts of *Rictor* and *Tsc2* in *Olig2-Cre* mice has shown that the developed technique is capable of discovering non-tuber white matter abnormalities, *e.g.* a significant reduction in the neurite density index (Figs. 11 and 12), in agreement with histological measurements (Fig. 13). Note, however, that for low myelinated and unmyelinated axons there might be significant exchange between the intra- and extra-neurite water pools, making the differentiation between hypomyelination and reduced axon density solely based on diffusion experiments not that straightforward. Our findings have clear translational significance as SMT may help oversee treatment success in promising clinical trials (Franz et al., 2006; Bissler et al., 2008; Krueger et al., 2010; Tillema et al., 2012) where patients receive mTOR inhibitors such as everolimus and sirolimus. Unlike the DTI-derived anisotropy metrics, the novel biomarkers are invariant with respect to the axon orientation distribution, which exhibits a high variability between subjects, and thus have potential to increase the detectability dramatically.

### Clinical translation

To establish the normative values of the microscopic diffusion indices, we have used high-quality data sets from a large cohort of healthy young adults acquired on a bespoke scanner (cf. Figs. 6 to 8). Our experiments have demonstrated that the acquisition time can be greatly reduced for rapid adoption in hospitals. Indeed, a moderate number of diffusion gradients evenly distributed over two *b*-shells is sufficient to recover the new biomarkers efficiently, as shown in Figs. 4 and 5. For example, a diffusion protocol with 30 gradient directions for each *b*-value of 1000 and 2500 s/mm^2^ – just twice as many as in standard DTI (Jones et al., 1999) – does not exceed 5 min of scan time on a today’s clinical MRI scanner when acquired with a multiband EPI sequence (Setsompop et al., 2012) for whole brain coverage in 2 mm isotropic resolution. As SMT is computationally very fast, microscopic diffusion maps can be made available to clinicians shortly after the scan has been finished. In conclusion, multi-compartment microscopic diffusion imaging has enabled us to reveal key features of brain microanatomy, such as neurite density, without unwanted side effects due to fibre crossings and orientation dispersion. The novel technique provides direct sensitivity to abnormalities in the microscopic tissue structure, as demonstrated in a model of tuberous sclerosis, and offers unique opportunities for various applications, ranging from clinical diagnostics to early patient stratification and treatment response assessment in interventional trials. Moreover, this framework recovers the neurite orientation distribution completely, which allows us to track crossing fibre pathways and then to quantify neural connectivity in the individual brain.

## Software

The software is available online at https://ekaden.github.io.

## Appendix A. Extra-neurite water diffusion

In the following we shall compare two different approaches to modelling the diffusion process in the extra-neurite compartment. Specifically, NODDI (Zhang et al., 2012) assumes that extra-neurite water is in fast exchange across all axon and dendrite orientations within a voxel, which is, however, questionable. The microenvironment a diffusing water molecule covers during the observation time is typically orders of magnitudes smaller than the dimension of the voxel the measured signal comes from. In more detail, Zhang et al. (2012) model the extra-neurite diffusion signal $e_{b}^{\text{ext}}(g)$ for a given *b*-value and gradient direction *g* as

(A:1) $${\begin{array}{cc} e_{b}^{\text{ext}}(g)=\exp\left(-bg^{t}{\bar{D}}^{\text{ext}}g\right), & \;\bar{D} \end{array}}^{\text{ext}}=\int_{S^{2}}p(\omega )D^{\mathrm{ext}}(\omega )d\omega ,$$

where *p*(***ω***) denotes the neurite orientation distribution and $D^{\text{ext}}(\omega )=(\lambda_{\|}^{\text{ext}}-\lambda_{⊥}^{\text{ext}})\omega \omega^{t}+\lambda_{⊥}^{\text{ext}}I_{3}$ the extra-neurite diffusion tensor for a dendrite or axon with orientation *ω*. $\lambda_{\|}^{\text{ext}}$ and $\lambda_{⊥}^{\text{ext}}$ are the parallel and perpendicular diffusivities, respectively. In plain words, Eq. (A.1) averages the microscopic diffusion tensor over the microdomain orientation distribution, instead of averaging the microscopic diffusion signal from the microscopic diffusion tensor as done in SMT-based microscopic diffusion anisotropy imaging, to obtain the macroscopic diffusion signal at the voxel level.

Furthermore, the fast-exchange approach leads to certain model inconsistencies. Let us consider a high-resolution image consisting of *n* equally sized voxels with neurite orientation distributions *p_i_*(***ω***) for *i*= 1, …,*n*, where all other parameters are the same. The extra-neurite diffusion signal of voxel *i* reads $e_{b,i}^{\text{ext}}(g)=\exp(-bg^{t}{\bar{D}}_{i}^{\text{ext}}g)$. If we reduce the resolution to one big voxel retrospectively, the total signal takes the form

(A:2) $$e_{b}^{\text{ext}}(g)=\frac{1}{n}\sum_{i=1}^{n}\exp\left(-bg^{t}{\bar{D}}_{i}^{\text{ext}}g\right).$$

On the other hand, we next measure a low-resolution image with just one voxel. The total neurite orientation distribution is then $p(\omega )=1/n\sum_{i=1}^{n}p_{i}(\omega )$ and, according to Eq. (A.1), the extra-neurite diffusion signal reads

(A:3) $$e_{b}^{\text{ext}}(g)=\exp\left(-bg^{t}\left[\frac{1}{n}\sum_{i=1}^{n}{\bar{D}}_{i}^{\text{ext}}\right]g\right),$$

which may be rewritten as

(A:4) $$e_{b}^{\text{ext}}(g)={\left(\prod_{i=1}^{n}\exp\left(-bg^{t}{\bar{D}}_{i}^{\text{ext}}g\right)\right)}^{1/n}.$$

It is obvious that in general Eqs. (A.2) and (A.4) give rise to different signals, which is contradictory. For this reason, in addition to the observation that the diffusing water molecules sense only a small environment in the range of few micrometres which typically includes merely a fraction of the neurite orientations present in a voxel, we do not assume the fast-exchange regime for the extra-neurite compartment here.
